# Dietary Carbohydrates Modulate *Candida albicans* Biofilm Development on the Denture Surface

**DOI:** 10.1371/journal.pone.0064645

**Published:** 2013-05-30

**Authors:** Ivone Lima Santana, Letícia Machado Gonçalves, Andréa Araújo de Vasconcellos, Wander José da Silva, Jaime Aparecido Cury, Altair Antoninha Del Bel Cury

**Affiliations:** 1 Department of Dentistry I, School of Dentistry, Federal University of Maranhão, São Luis, Maranhão, Brazil; 2 Department of Prosthodontics and Periodontology, Piracicaba Dental School, University of Campinas, Piracicaba, São Paulo, Brazil; 3 Department of Physiological Sciences, Piracicaba Dental School, University of Campinas, Piracicaba, São Paulo, Brazil; Instituto de Salud Carlos III, Spain

## Abstract

The purpose of this study was to investigate whether dietary carbohydrates can modulate the development of *Candida albicans* biofilms on the denture material surface. Poly (methyl methacrylate) acrylic resin discs were fabricated and had their surface roughness measured. Biofilms of *C.* *albicans* ATCC 90028 were developed on saliva-coated specimens in culture medium without (control) or with carbohydrate supplementation by starch, starch+sucrose, glucose, or sucrose for 72 h. The cell count, metabolic activity, biovolume, average thickness, and roughness coefficient were evaluated at the adhesion phase (1.5 h) and after 24, 48, and 72 h. The secretion of proteinases and phospholipases, cell surface energy, and production of extra/intracellular polysaccharides were analyzed after 72 h of biofilm development. Data were analyzed by one- and two-way ANOVA followed by Tukey’s test at 5% significance level. In the early stages of colonization (adhesion and 24 h), the glucose group showed the highest cell counts and metabolic activity among the groups (*p*<0.05). After maturation (48 and 72 h), biofilms exposed to glucose, sucrose, or starch+sucrose showed higher cell counts and metabolic activity than the control and starch groups (*p*<0.001). Compared to the control group, biofilms developed on starch or starch+sucrose had more proteinase activity (*p*<0.001), whereas biofilms developed on glucose or sucrose had more phospholipase activity (*p*<0.05). Exposure to starch+sucrose increased the production of extracellular and intracellular polysaccharides (*p*<0.05). Biofilms developed on starch or without carbohydrate supplementation presented cells with more hydrophobic behavior compared to the other groups. Confocal images showed hyphae forms on biofilms exposed to starch or starch+sucrose. Within the conditions studied, it can be concluded that dietary carbohydrates can modulate biofilm development on the denture surface by affecting virulence factors and structural features.

## Introduction

The availability of nutrient sources plays an important role in the pathogenesis of fungal infections [1], [2]. Carbohydrates consumed in the diet are the primary and preferred nutrient sources for *Candida albicans* [3]–[5], the main pathogen related to *Candida*-associated denture stomatitis (CADS) [6]. Additionally, denture wearers tend to choose a carbohydrate-rich diet because of difficulty in chewing [7]. The constant supply of sugars such as glucose, sucrose, and starch may create an environment conductive to *C. albicans* colonization [3], [5], [8]. Thus, understanding how *C. albicans* biofilms behave under exposure to dietary carbohydrates is of outmost relevance, although little data are available so far.

The first step in the dynamics of CADS pathogenesis is the adhesion of *C. albicans* to the denture surface and epithelial cells, followed by cell multiplication, organization, and secretion of the extracellular matrix (ECM). This process results in the formation of a three-dimensional (3D) structure known as biofilm [9]. During biofilm formation, the abilities of cells to adhere and co-aggregate are influenced by the cell surface hydrophobicity, an important virulence factor [10]. In this respect, it is possible that exposure to dietary carbohydrates could alter the components of fungal cells, affecting their hydrophobicity [8], [11], [12] and, consequently, their capacity for biofilm formation [5]. Additionally, the production of organic acids during carbohydrate hydrolysis [13] could activate other virulence factors, such as the yeast-hyphae transition and the secretion of hydrolytic enzymes (*e.g*., phospholipases and proteases) [10], [14]. These virulence factors are mainly related to the invasion of host tissues, which aggravates the inflammatory response of the oral mucosa [15], [16].

Also, microbial communities adapt to environmental conditions by altering their structural organization [17]. Thus, it is expected that dietary carbohydrates could provoke structural changes in *C. albicans* biofilms. Fermentable carbohydrates, such as glucose, sucrose, and starch, have been observed to serve as substrates for ECM synthesis in bacterial biofilm models [18]. Additionally, sucrose and starch are consumed simultaneously in modern societies [19]. This combination could enhance the pathogenicity of biofilms by altering the organization of cells and leading to the formation of a structurally distinct ECM relative to other carbon sources [20]. However, it remains unclear how these carbohydrates affect the structure of *C. albicans* biofilms.

Thus, this study investigated whether dietary carbohydrates or their combinations can modulate the development of *C. albicans* biofilms on the surface of denture material.

## Materials and Methods

### Study Design

This *in vitro* study had a randomized and blinded design. Discs were fabricated using a water bath poly (methyl methacrylate) (PMMA) acrylic resin. The surface roughness of all specimens and the surface free energy for the salivary-coated specimens were measured. Biofilms of *C. albicans* ATCC 90028 were developed on saliva-coated specimens in culture medium without or with carbohydrate supplementation by starch, starch+sucrose, glucose, or sucrose for 72 h. The cell count, metabolic activity, biovolume, average thickness, and roughness coefficient were evaluated at the adhesion phase (1.5 h) and after 24, 48, and 72 h of biofilm development. The secretion of proteinases and phospholipases, cell surface energy, and production of extra/intracellular polysaccharides were analyzed after 72 h of biofilm development. All of the experiments were performed in four replicates of three independent experiments on different days (*n* = 12). Data were analyzed by one-way and two-way ANOVA followed by Tukey’s test at the 5% significance level.

### Fabrication of Specimens

Discs (10-mm diameter, 2-mm thickness) were fabricated using a water bath PMMA acrylic resin (QC-20; Dentsply Ltd., Weybridge, England) according to manufacturers’ directions (25±1°C and 50% ±5% relative humidity). Prior to polymerization for 20 min at 100°C, the resin mass in the plastic phase was packed into a metal mold to standardize the specimen dimensions. Processed specimens were immersed in distilled water for 48 h at 35°C to release residual monomer [21]. To simulate the inner side of a denture, the specimen surfaces were ground in an horizontal polisher (model APL-4; Arotec, São Paulo, Brazil) by using progressively smoother aluminum oxide papers (320, 400, and 600 grit). The surface roughness of all of the specimens was analyzed by a profilometer (Surfcoder SE 1700; Kosaka Laboratory Ltd., Kosaka, Japan) accurate to the nearest 0.01 µm and calibrated at a specimen length of 0.8 mm, 2.4 mm percussion of measure, and 0.5 mm/s. The mean of three measurements for each specimen was calculated, and the surface roughness was standardized at 0.30±0.04 µm [22]. The surface free energy of the saliva-coated specimens was measured with a goniometer (Ramé-Hart Instrument Co., NJ, USA) by an acid-base technique [23]. The total surface free energy was 40.05±1.00 mJ/m^2^.

After these measurements had been made, the specimens were ultrasonically cleansed (Thornton T 740; Thornton-Inpec Eletronica Ltd., Vinhedo, São Paulo, Brazil) in purified water for 20 min to remove any contaminants and artifacts.

### Preparation of Candida Suspensions

*C. albicans* (ATCC 90028, reference strain) was aerobically cultured from original broth by incubation in Sabouraud Dextrose Agar (SDA; Difco, Detroit, MI, USA) for 24 h at 35°C. A loop of yeast cells was inoculated into Yeast Nitrogen Base culture medium (YNB; Difco) supplemented with 50 mM glucose and incubated aerobically under agitation at 35°C. During the exponential growth phase (*i.e.*, after 18–20 h of incubation), the *C. albicans* cells were washed twice with phosphate-buffered saline (PBS; pH 7.2). Suspensions were prepared in YNB medium without (control group) or with carbohydrate supplementation by 1% starch (starch group), 1% starch +1% sucrose (starch+sucrose group), 1% glucose (glucose group), or 1% sucrose (sucrose group). The suspensions were standardized spectrophotometrically (Spectronic 20; Bausch & Lomb, Rochester, NY, US) to ∼10^7^ cells/mL (OD = 0.25 at 520 nm).

### Biofilm Development

To mimic the oral cavity, the specimens were coated with human salivary pellicle prior to biofilm development. Saliva was collected from a healthy volunteer, who provided written formal consent according to a protocol approved by the Ethics Committee in Research of Piracicaba Dental School (105/2011). Saliva was collected in an ice-chilled polypropylene tube during masticatory stimulation with a flexible film and clarified by centrifugation (10,000×*g* for 5 min at 4°C). The supernatant was filter-sterilized and used immediately.

Under aseptic conditions, each specimen was placed in a presterilized 24-well culture plate. Two milliliters of saliva were added to each well. The plate was incubated aerobically under agitation for 1 h at 35°C to form the salivary pellicle. Saliva-coated specimens were transferred to a 24-well culture plate, and the prepared standard suspensions were added to each well. The sets were incubated aerobically under agitation at 35°C for 1.5 h (adhesion phase). The specimens were carefully washed with PBS and transferred to new presterilized 24-well culture plates containing culture medium without or with carbohydrate supplementation. At the end of each 24-h period, the specimens were washed with PBS, and fresh medium was added. The plates were incubated aerobically under agitation for 72 h at 35°C.

### Cell Count

Biofilm-containing specimens were washed twice with PBS, and, afterwards, were immersed in PBS, and sonicated (7 W, for 30 s) to disrupt the biofilm structure. The sonicated solutions were serially diluted in PBS, and samples were plated in triplicate onto SDA. The plates were incubated aerobically for 24 h at 35°C. Yeast cells were counted (in cells/mL) on a stereomicroscope (Coleman Comp. Imp., Santo André, São Paulo, Brazil).

### Metabolic Activity

The metabolic activity was determined with an adapted XTT assay [24]. Briefly, biofilm-containing specimens were placed in a 24-well culture plate with XTT solution (PBS supplemented with 200 mM glucose, 1 mg/mL XTT, and 0.4 mM menadione). The plates were protected from light and incubated under agitation for 3 h at 35°C. Colorimetric changes in the supernatant were analyzed spectrophotometrically at 492 nm.

### Secretion of Proteinase and Phospholipase

Enzyme secretion assays were performed on sonicated suspensions of biofilm cells only after 72 h of biofilm development because of the large amounts of biofilm that were needed for these analyses. The proteinase activity was determined as described previously [25]. Briefly, the supernatant was mixed with 1% azocasein at 1∶9 (v/v) for 1 h at 35°C. The reaction was stopped by adding 500 µL of 10% trichloroacetic acid. The mixture was centrifuged at 10,000×*g* for 5 min. Next, 500 µL of the supernatant were mixed with an equal volume of 0.5 M NaOH and incubated for 15 min. The proteinase activity was evaluated spectrophotometrically at 440 nm. The specific proteinase activity was defined as the amount of enzyme that elicited an increase of 0.001 units of absorbance per minute of digestion by biofilm dry weight [25], [26].

The phospholipase activity was determined as described by Taniguchi et al. [27]. Briefly, the biofilm supernatants were mixed and incubated with an equal volume of phosphatidylcholine substrate for 1 h at 35°C. The phospholipase activity was evaluated spectrophotometrically at 630 nm. The specific phospholipase activity was established as the absorbance shift per minute of reaction by biofilm dry weight [26], [27].

### Cell Surface Energy

The cell surface energy was evaluated after 72 h of biofilm development because of the large amount of biofilm that was needed for this analysis. Biofilm-containing specimens were washed twice, immersed in PBS, and sonicated to disrupt the biofilm structure. The sonicated solution was filtered through a 0.45-µm cellulose acetate membrane, which had been previously soaked with 10 mL of distilled water, to obtain a homogeneous cell layer. The filters with cell layers were placed on a glass slide and maintained in a Petri dish containing 1% (w/v) agar and 10% (v/v) glycerol for 3 h at 35°C [28].

The cell surface energy was determined by an acid-base technique [23] with goniometer (Ramé-Hart Instrument Co.). Briefly, droplets (15 µL) of distilled water, formamide, and 1-bromonaphthalene were dispensed on the filters with cell layers. The contact angle was automatically measured. The surface free energy was measured by the DropImage software package (Ramé-Hart Instrument Co.), which allowed the hydrophobicity of fungal cells to be estimated.

### Polysaccharide Extraction

For polysaccharide extraction, 400 µL of the previously obtained sonicated biofilm suspension were centrifuged at 10,000×g for 5 min at 4°C. The supernatant, which contain the soluble extracellular polysaccharides (SEPs), was transferred to a tube named SEPs. An aliquot of 1 M NaOH was added to the pellet to extract the insoluble extracellular polysaccharides (IEPs) [29]. The tube was agitated for 15 min, centrifuged, and the supernatant was transferred to another tube named IEPs. Finally, an aliquot of 1 M NaOH was added to the residual pellet to extract the intracellular polysaccharide (IPs) [30]. The tube was heated for 15 min at 100°C, centrifuged, and the supernatant was transferred to another tube named IPs.

Three volumes of cold ethanol were added to the tubes containing SEPs, IEPs, and IPs. The tubes were maintained for 30 min at −20°C. Next, the tubes were centrifuged, and the pellets were washed twice with cold 75% ethanol. The precipitated polysaccharides were resuspended in 1 M NaOH. The total carbohydrate was estimated by the phenol sulfuric method [31]. The results were normalized by dry weight of biofilm.

### Confocal Structural Analysis

The biofilm structure was evaluated at the adhesion phase and after 24, 48, or 72 h of biofilm development by confocal laser-scanning microscopy (CLSM) (Leica Microsystems CMS, Mannheim, Germany). Biofilm-containing specimens were washed twice with PBS and stained by SYTO-9 and propidium iodide with the Live/Dead *BacLight* viability kit (Invitrogen-Molecular Probes, Eugene, OR, USA). The biofilms were incubated for 20 min at 35°C, with care taken to protect the samples from light. A series of optical sections was taken by CLSM through the full depth of the biofilm at 1-µm intervals in the *z*-axis. At least five representative random optical fields were examined for each specimen. The resulting series of images was analyzed by the COMSTAT software program [17] to quantify structural parameters, such as the biovolume (µm^3^/µm^2^), average thickness (µm), and roughness coefficient (µm).

### Statistical Analysis

The results were statistically analyzed by the SAS/LAB software package (SAS Software, version 9.0; SAS Institute Inc., Cary, NC, USA). Assumptions of the equality of variances and the normal distribution of errors were checked. Data were transformed as suggested by the software, as follows: cell counts, IEPs, and IPs (transformed by log 10); metabolic activity, phospholipase and proteinase activities, and SEP (transformed by exponentiation, y^2^), average thickness (transformed by exponentiation, y^3^), and roughness coefficient (transformed by exponentiation, y^5^).

The proteinase and phospholipase activities, cell surface energy, and polysaccharide production were analyzed by one-way ANOVA followed by Tukey’s HSD test, considering the carbohydrates or their combination as the study factor. The cell count, metabolic activity, biovolume, average thickness, and roughness coefficient were analyzed by two-way ANOVA followed by Tukey’s HSD test. In this case, the time-point of biofilm development (adhesion, 24, 48, or 72 h) and the carbohydrates or their combination were considered as study factors.

## Results

**Figure 1:**
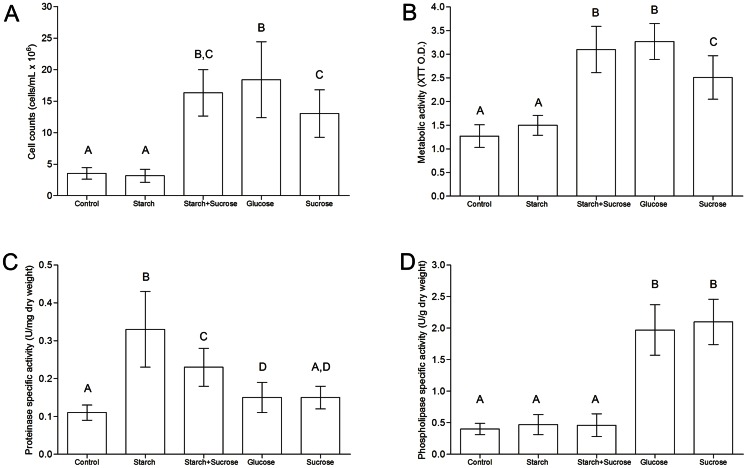
*C. albicans* biofilms developed under exposure to dietary carbohydrates. (A) Cell count and (B) metabolic activity of mature biofilms of *C. albicans* (48 and 72 h). (C) Proteinase-specific activity and (D) phospholipase-specific activity of *C. albicans* biofilms (72 h).

No statistically significant differences were observed between the adhesion phase and 24 h, or between 48 and 72 h for any of the parameters investigated during biofilm development (*p*>0.05). However, during the early stages of colonization (adhesion phase and 24 h of development), the glucose group showed the highest cell counts and metabolic activity among the groups (*p*<0.05). No differences were observed among the other groups at these time points (*p*>0.05, data not shown). Similarly, for the mature biofilms (48 and 72 h of development), glucose, sucrose, or starch+sucrose exposure led to higher cell counts and metabolic activity compared to starch or no carbohydrate exposure (*p*<0.001). Mature biofilms developed in the presence of starch showed cell counts and metabolic activity that were similar to those of the control group during these experimental periods (*p*>0.05, Fig. 1A, B).

Biofilms developed on starch secreted the highest amount of proteinase (*p*<0.001), followed by biofilms developed on starch+sucrose (Fig. 1C). The phospholipase activity was higher in the glucose and sucrose groups compared to the control, starch, and starch+sucrose groups (*p*<0.05, Fig. 1D).

**Figure 2:**
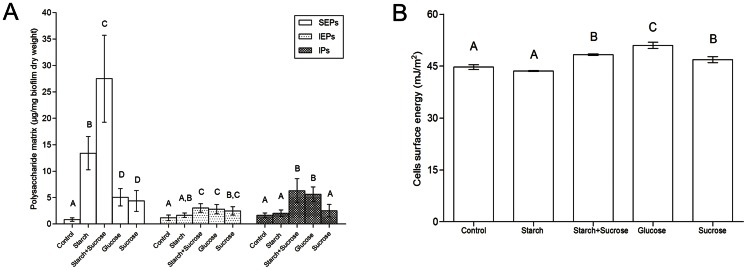
*C. albicans* biofilms (72 h) developed under exposure to dietary carbohydrates. (A) Polysaccharide matrix and (B) cell surface energy.

The starch+sucrose group showed the highest production of SEPs followed by the starch group (*p*<0.05, Fig. 2A). No significant difference in SEPs production was found between the glucose and sucrose groups, which both presented greater SEPs amounts than the control group (*p*<0.001). The IEPs production was similar among biofilms exposed to starch+sucrose, sucrose, and glucose (*p*>0.05). The starch+sucrose and glucose groups produced the highest amounts of IPs (*p*<0.05, Fig. 2A).

The cell surface energy varied depending on the carbohydrate source. The glucose group displayed cells with the highest hydrophilicity (*p*<0.05). The starch and control groups resulted in cells with more hydrophobic behaviors (*p*<0.05, Fig. 2B).

**Figure 3:**
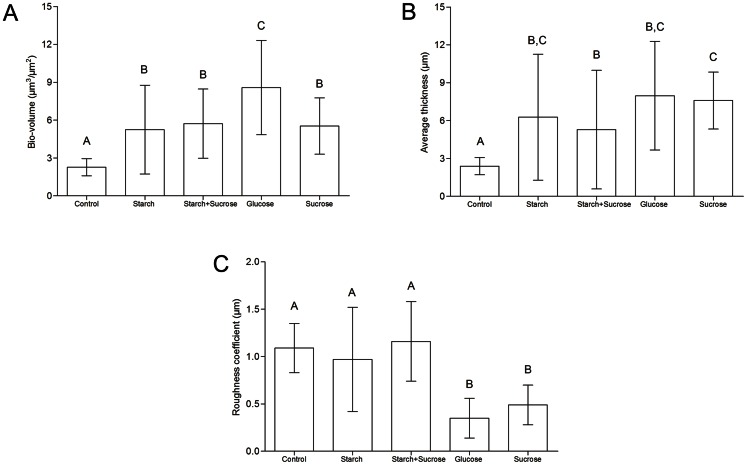
Structural parameters of *C. albicans* biofilms (48 and 72 h) developed under exposure to dietary carbohydrates. (A) Biovolume, (B) average thickness, and (C) roughness coefficient.

Regarding the structural parameters obtained by CLSM images, during the early stages of colonization, no differences in the biovolume, average thickness, and roughness coefficient were observed between the groups (*p*>0.05, data not shown). Also, biofilms developed for 48 or 72 h did not differ from each other, but they differed from the early-stage biofilms. Mature biofilms exposed to glucose presented the highest biovolume (*p*<0.05, Fig. 3A). The control group showed the lowest biovolume and average thickness values (*p*<0.001, Fig. 3A, B). The glucose and sucrose groups showed the lowest roughness coefficient values (*p*<0.05, Fig. 3C).

**Figure 4:**
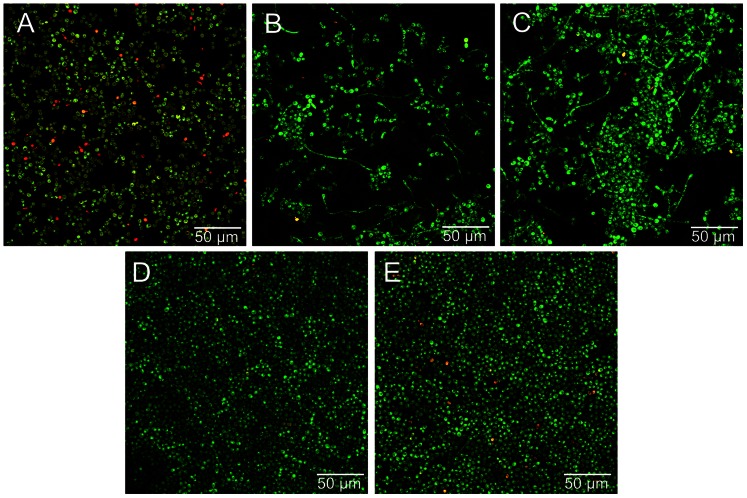
Representative confocal images of *C. albicans* biofilms (72 h) developed under exposure to dietary carbohydrates. (A) Control, (B) starch, (C) starch+sucrose, (D) glucose, and (E) sucrose. Live cells appear green and dead cells appear red. A considerable number of hyphae forms can be observed in images B and C.

Figure 4 shows representative CLSM images of *C. albicans* biofilms at 72 h. These images reveal the presence of black spaces and hyphae forms on biofilms developed on starch (Fig. 4B) or starch+sucrose (Fig. 4C). In addition, higher amounts of dead cells can be observed on the control (Fig. 4A) and starch (Fig. 4B) groups compared to the other groups.

## Discussion

In this study, we investigated whether dietary carbohydrates or their combinations could modulate the development of *C. albicans* biofilms by affecting the cell count, metabolic activity, proteinase and phospholipase secretion, hydrophobicity, polysaccharide production, and structural features.

During the early stages of biofilm development, the cell count and metabolic activity were significantly increased in the presence of glucose. This result may be explained by considering that when yeast cells are colonizing a surface and are exposed to different carbon sources, they preferentially utilize monosaccharides, such as glucose, before assimilating others carbon sources [32]. For the mature biofilms, the glucose, starch+sucrose, and sucrose groups showed the highest cell counts and metabolic activity. After achieving an advanced stage of organization and metabolism, it may be that the yeast cells are able to hydrolyze more complex carbon sources, such as sucrose, which could be a critical factor during biofilm formation [33]. This hierarchical mechanism of carbon assimilation is regulated by a complex glucose-signaling network, which involves the coordinated action of AMP kinase, protein kinase A, and sugar repressor signaling pathways [34].

Besides glucose and sucrose, starch is commonly found in a carbohydrate-rich diet [19]. Although starch is classified as a polysaccharide and can be a good nutrient source, our results suggested that it was not easily degraded by yeast cells. Actually, starches are rapidly hydrolyzed into maltose, maltodextrins, and other oligosaccharides by salivary α-amylase in the oral environment [20]. Although our biofilms were developed on a salivary pellicle, the salivary pellicle did not appear to be sufficient to hydrolyze the starch. Consequently, the exposure to starch resulted in lower cell counts and metabolic activity during all experimental periods, which were similar to levels of the control group.

In this study, it is possible to suggest that the control and starch groups represented nutrient-limited conditions. These environmental conditions resulted in lower structural bases and higher amounts of dead cells, as clearly seen in the CLSM images. Whereas the biofilms developed in the presence of glucose, sucrose, and starch+sucrose formed robust 3D structures, the presence of nutrient-limited conditions resulted in negligible biofilm formation. This behavior may be attributed to a possible “starved” condition, in which the metabolic capacity of *C. albicans* may have been greatly depressed and the growth and proliferation inhibited [35]. This possibility was confirmed by our cell count, metabolic activity, biovolume, and average thickness results.

Biofilm dispersion could also be induced under nutrient-limited conditions [36]. In this regard, the high roughness coefficient shown by the “starved” groups by COMSTAT analysis can be considered as an indicator of heterogeneity [17], reflecting their irregularity and dispersion on the substratum surface. Overall, it is supposed that these metabolic and structural changes are beneficial for survival under nutrient-limited conditions. Future investigations should consider the exact molecular mechanisms that allow fungal cells to survive in the starvation environment.

The adhesion of *Candida* to the denture surface and the coaggregation between cells are critical virulence factors for biofilm formation [9], [10]. Dietary sugars may affect adherence by altering the components of the fungal cell wall [5], [8], [11], [12]. *C. albicans* possesses many cell-wall proteins [37] that vary considerably both in quantity and quality under different environmental conditions [1], [2], [35]. Because these cell-wall proteins are closely related to the cell-surface properties, profound changes are expected to be observed in the hydrophobicity of fungal cells when they are exposed to different carbohydrate sources [38].

In our data, biofilms exposed to starch or no carbohydrate had more hydrophobic behavior. In contrast, glucose exposure, followed by sucrose and starch+sucrose exposure, resulted in cells that were more hydrophilic. These data support the idea that the lowest biofilm formation occurred under the “starved” conditions. A previous study demonstrated that the salivary pellicle coating the denture surface had many hydrophilic proteins [39]. In addition, the adherence and biofilm development of hydrophilic species (*e.g.*, *C. albicans*) are known to be increased on more hydrophilic surfaces [40], [41]. Thus, changes in the cell-wall proteins under nutrient-limited conditions may account for the higher hydrophobicity and, consequently, reduced the biofilm formation.

*C. albicans* produces numerous short-chain carboxylic acids during carbohydrate hydrolysis [13], which are related to the reduction in the environmental pH [42]. This condition may activate other virulence factors, such as the yeast-hyphae transition and the secretion of proteinases and phospholipases [10], [14]. According to the CLSM images, biofilms developed in glucose, sucrose, or without carbohydrate supplementation were predominantly composed of only blastospore cells, whereas the starch and starch+sucrose groups also displayed a filamentous growth mode. Interestingly, the biofilms with hyphae also showed the highest proteinase activity. A previous study demonstrated that some proteinase isoenzymes were preferentially expressed during hyphae formation [16], which may help to explain our results.

On the other hand, the phospholipase activity was greater when biofilms were exposed to glucose or sucrose. It is possible that this secretion was modulated by environmental conditions, such as the pH of the medium [15], rather than by morphological aspects. Phospholipases are more active in pH ranging from 2.5 to 3.5 [43]. The pH condition observed in the glucose and sucrose groups after biofilm maturation (pH ∼3.2, data not shown) was lower than that of the other groups.

Our biochemical analyses demonstrated that the presence of starch+sucrose resulted in the highest amounts of soluble and insoluble polysaccharides, consistent with previous reports using bacterial biofilm models [18]. Additionally, the energetic reserve represented by the IPs was also increased for this group. A previous study showed that the proportion of β-glucans and mannoproteins, the main fungal matrix components, could vary depending on the nature of the fermentative carbon source [44]. This result seems to be very important, given that the ECM creates a 3D environment that is important for both the biofilm integrity and its resistance to antifungal agents [9]. It may be that the combination of starch+sucrose can trigger *C. albicans* responses at the transcriptional level [20], modulating the expression of genes associated with ECM formation. Nevertheless, the exact mechanism by which dietary carbohydrates and their combinations enhance ECM production by *C. albicans* biofilms requires future investigation.

### Conclusion

Within the limitations of this study, it can be concluded that dietary carbohydrates can modulate biofilm development on the denture surface by affecting virulence factors and structural features.
